# Comparison of Rhenium and Iodine as Contrast Agents in X-Ray Imaging

**DOI:** 10.1155/2021/1250360

**Published:** 2021-11-01

**Authors:** José Carlos De La Vega, Pedro Luis Esquinas, Jovan Kaur Gill, Selin Jessa, Bradford Gill, Yogesh Thakur, Katayoun Saatchi, Urs O. Häfeli

**Affiliations:** ^1^Faculty of Pharmaceutical Sciences, University of British Columbia, Vancouver, British Columbia, Canada; ^2^Medical Imaging Research Group, Department of Radiology, University of British Columbia, Vancouver, British Columbia, Canada; ^3^Medical Physics Department, BC Cancer, Vancouver, British Columbia, Canada; ^4^Department of Radiology, University of British Columbia, Vancouver, British Columbia, Canada; ^5^Medical Imaging, Vancouver Coastal Health, Vancouver, British Columbia, Canada

## Abstract

**Purpose:**

The majority of X-ray contrast agents (XCA) are made with iodine, but iodine-based XCA (I-XCA) exhibit low contrast in high kVp X-rays due to iodine's low atomic number (*Z* = 53) and K-edge (33.1 keV). While rhenium is a transition metal with a high atomic number (*Z* = 75) and K-edge (71.7 keV), the utilization of rhenium-based XCA (Re-XCA) in X-ray imaging techniques has not been studied in depth. Our study had two objectives: (1) to compare both the image quality and the absorbed dose of I- and Re-XCA and (2) to prepare and image a rhenium-doped scaffold. *Procedures*. I- and Re-XCA were prepared and imaged from 50 to 120 kVp by Micro-computed tomography (*µ*CT) and digital radiography and from 120 to 220 kVp by planar X-ray imaging. The scans were repeated using 0.1 to 1.6 mm thick copper filters to harden the X-ray beam. A rhenium-doped scaffold was prepared via electrospinning, used to coat catheters, and imaged at 90 kVp by *µ*CT.

**Results:**

I-XCA have a greater contrast-to-noise ratio (CNR) at 50 and 80 kVp, but Re-XCA have a greater CNR at >120 kVp. The difference in CNR is increased as the thickness of the copper filters is increased. For instance, the percent CNR improvement of rhenium over iodine is 14.2% with a 0.6 mm thick copper filter, but it is 59.1% with a 1.6 mm thick copper filter, as shown at 120 kVp by *µ*CT. Upon coating them with a rhenium-doped scaffold, the catheters became radiopaque.

**Conclusions:**

Using Monte Carlo simulations, we showed that it is possible to reduce the absorbed dose of high kVp X-rays while allowing the acquisition of high-quality images. Furthermore, radiopaque catheters have the potential of enhancing the contrast during catheterizations and helping physicians to place catheters inside patients more rapidly and precisely.

## 1. Introduction

X-ray imaging techniques require the administration of X-ray contrast agents (XCA) to enhance the contrast between fluid, tissue, and/or anatomical structures. The majority of XCA are solutions of iodinated compounds, which are administered to patients via intravenous or intra-arterial injections before radiographic examinations [1]. The evolution in the structure of iodine-based XCA (I-XCA) moved from inorganic iodine (specifically sodium iodide, or NaI) to organic mono-, di-, and tri-iodinated compounds; from lipophilic to hydrophilic compounds; from ionic to nonionic compounds; and more recently, from monomers to dimers. These modifications aimed to increase the iodine content and reduce the incidence of adverse effects [2].

I-XCA are rapidly excreted by the kidneys, thus requiring repeated large doses to achieve good contrast. The dose of iodine is typically small, ∼3 g in lumbar, thoracic, cervical, and columnar radiographic examinations [3, 4], but it could be as high as ∼30 g in coronary computed tomography (CT) angiography [5] and ∼100 g in angiography and angioplasty [6]. I-XCA have been linked to several adverse effects with an estimated incidence between 1% and 12% [7]. These adverse effects may range from mild reactions, such as itching and emesis, to life-threatening emergencies, such as hypersensitivity reactions, thyroid dysfunction, anaphylaxis, and nephropathy [8–10].

At the energies and atomic numbers under consideration, the attenuation of X-rays is dependent upon the atomic number raised to the third power [11]. Due to its low atomic number (*Z* = 53), iodine attenuates high kVp X-rays (>80 kVp) to a lesser degree than other high-*Z* elements. For this reason, images typically exhibit low contrast and high noise [12, 13]. While most scans are performed between 24 (e.g., mammography) and 140 kVp (e.g., chest CT) [14], the suboptimal attenuation of iodine may be problematic in scans of average-sized and large patients, which are typically performed at 100 to 140 kVp [15–17].

The development of iodine-free XCA has garnered a lot of interest to overcome these limitations. Most research has focused on replacing iodine with elements with a greater atomic number, such as gold (*Z* = 79) [18–22], bismuth (*Z* = 83) [23, 24], and tantalum (*Z* = 73) [25, 26]. Among all these elements, gold has played the leading role in the development of iodine-free XCA, in the form of nanoparticles (NP). One of the advantages of gold NP (AuNP) is that they have been investigated for many years for other biomedical applications (e.g., photoacoustic imaging, photothermal ablation, and DNA detection) [27]. AuNP have been shown to exhibit good biotolerability (Median Lethal Dose, or LD_50_ = 3.2 g of gold per kg) [21, 28, 29]. Many researchers have examined the X-ray attenuation of AuNP through both phantom and animal studies. These studies have shown that AuNP have a higher X-ray attenuation [18–21] and a longer circulation half-life [30–32] than I-XCA.

Since gold is more expensive than iodine, other high-Z elements, like bismuth and tantalum, have also been studied. Bismuth has the advantage of decomposing *in vivo* to small and renally clearable Bi(III) species as a result of its tendency to oxidize and hydrolyze in water (i.e., it does not accumulate in the body) [33]. A few examples of bismuth NPs (i.e., BiNPs) under investigation are ultra-high payload BiNPs [34], bismuth glyconanoparticles (BiGNP) [35], and bismuth(III) sulfide (Bi_2_S_3_) NP [23, 24]. Tantalum is also of interest as an XCA, particularly when it reacts with oxygen to form tantalum pentoxide (or Ta_2_O_5_) due to its biocompatibility, chemical stability, and high solubility in water. The use of tantalum pentoxide as an XCA was reported many years ago in bronchography, after delivery as a powder aerosol via the trachea [36]. Current efforts revolve around the production of NP comprised of a core made of tantalum pentoxide [25, 26].

The utilization of rhenium in X-ray imaging has only been reported in a few manuscripts by Krasilnikova et al. [37–40] despite its high atomic number (*Z* = 75) and low cost compared with gold (1,300 USD per kg of rhenium vs. 44,800 USD per kg of gold) [41]. Rhenium is a chemically versatile element with rich coordination chemistry. While it can exist in oxidation states from −1 to +7 [42], rhenium complexes are thermodynamically more stable in their higher oxidation states and they oxidize *in vivo* to perrhenate (ReO_4_−). This transformation occurs because perrhenate features rhenium in the oxidation state of +7, which is rhenium's preferred oxidation state [42, 43]. This is of importance for clinical applications because the pharmacokinetics of perrhenate is well-understood: it is taken up by the thyroid, retained there with a biological half-life of 12 h, and finally excreted via the kidneys [44–47]. Furthermore, perrhenate has been shown to have a half-maximal effective concentration (EC_50_) of 1 mM or higher, which is an indication of its low toxicity [48, 49].

The objective of this study was to compare the attenuation of Re- and I-XCA over a wide range of experimental conditions. We evaluated the image quality, which was calculated experimentally using preclinical and clinical equipment, and the absorbed dose, which was estimated through Monte Carlo simulations for clinical equipment. Specifically, the samples were imaged from 50 to 120 kVp by micro-CT (*µ*CT) (preclinical equipment) and digital radiography (clinical equipment) and from 120 to 220 kVp by planar X-ray imaging (preclinical equipment).

A specific application of the use of rhenium in X-ray imaging was studied by coating catheters with a rhenium-doped scaffold. Large doses of I-XCA are administered to patients during catheterizations because the majority of catheters are radiolucent. The incorporation of a rhenium-doped scaffold onto the surface of the catheters has the potential of making them radiopaque, thus providing a visual guide for physicians during catheterizations and potentially reducing the use of I-XCA.

## 2. Material and Methods

### 2.1. Investigation of the Image Quality

Re- and I-XCA with a final concentration of rhenium and iodine of 50, 100, and 200 mM were prepared by diluting ammonium perrhenate (Sigma-Aldrich; Oakville, Ontario, Canada) and iohexol (Omnipaque™, 300 mg of I per mL, GE Healthcare; Little Chalfont, Buckinghamshire, United Kingdom), which is a clinically used I-XCA, in water.

#### 2.1.1. Micro-Computed Tomography

Six microcentrifuge tubes with 1 mL of the Re- and I-XCA were prepared. Two additional microcentrifuge tubes with water and air were prepared as controls. The microcentrifuge tubes were placed in a cylindrical phantom (mCTP 610, Shelley Medical Imaging Technologies; London, Ontario, Canada), where they were arranged in a concentric circle. The phantom was made of three poly(methyl methacrylate) (PMMA, or Plexiglas®) plates contained in a polycarbonate housing. The thickness of each plate was 1.3 cm, thus providing a total filtration of 3.9 cm of PMMA [50].

The XCA were imaged using a preclinical *µ*CT scanner (eXplore CT120, TriFoil Imaging; Chatsworth, California, United States) with an inherent filtration of 1 mm of aluminum and 0.15 mm of beryllium. As additional filtration, a 0.6 mm thick copper filter was placed between the phantom and the X-ray tube. The settings were set at 50 kVp (80 mAs and 125 ms), 80 kVp (60 mA and 125 ms), and 120 kVp (40 mA and 63 ms).

To evaluate the effect of additional filtration on the image quality, the 120 kVp scan was repeated with 1.2 and 1.6 mm thick copper filters between the phantom and the X-ray tube. SpekCalc, a computational tool available in MATLAB™ [51, 52], was utilized to predict the X-ray spectra for all the exposures performed at 120 kVp.

The settings of all the scans are summarized in Table 1.

The acquisition consisted of 1,440 projections per scan. The scanner was operated in a step-and-shoot mode. To cool the anode during acquisition, a step delay of 3 s was used for the 50 and 80 kVp scans, while a step delay of 7 s was used for the 120 kVp scans.

MicroView (Parallax Innovations; Ilderton, Canada) was used to reconstruct the data as three-dimensional (3D) images with a matrix size of 1455 × 1455 and a spatial resolution of 50 *µ*m. For each 3D image, this software was also utilized to measure the mean voxel intensity of each sample in a cylindrical volume of interest (VOI) with a diameter and height of 50 pixels. The contrast-to-noise ratio (CNR) of each sample was calculated relative to water using the following equation:

(1) $$\begin{array}{c} \text{CNR}=\frac{{\bar{\iota }}_{i}-\;{\bar{\iota }}_{H_{2}O}\;}{\sigma_{H_{2}O}}, \end{array}$$

where $\bar{\iota }$ represents the mean voxel intensity, *σ* represents the standard deviation of the mean voxel intensity, and *i* represents the sample of interest [53]. The analysis was conducted in triplicate.

#### 2.1.2. Planar X-Ray Imaging

The XCA with a concentration of rhenium and iodine of 200 mM were imaged between 120 and 220 kVp using the Small Animal Radiation Research Platform (SARRP) (Xstrahl, Walsall Wood, UK). The XCA were imaged in microcuvettes with 1 mL each. Two microcuvettes with water and air were included in all the scans as controls. The XCA were arranged in a straight line 30 cm away from the X-ray tube. A 0.1 mm thick copper filter was used as additional filtration. The settings of all the scans are summarized in Table 2.

**Figure 1:**
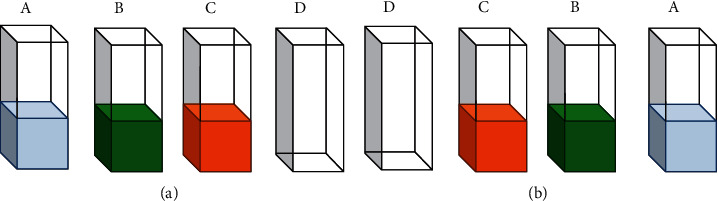
Setup for planar X-ray imaging. The Re- and I-XCA were imaged initially as indicated in (a) (“original” configuration). Then, they were rearranged as indicated in (b) (“inverted” configuration) and imaged again. The images from both configurations at each X-ray tube potential were averaged to generate a single image, which was then utilized to calculate the CNRs. The image shows (A) water, (B) Re-XCA, (C) I-XCA, and (D) air.

To minimize the heel effect, which causes discrepancies in the intensity of the pixels from the left to the right side of the images as a result of the reduction in the intensity of the X-ray beam along the cathode-anode axis in the X-ray tube, the position of the XCA was changed between scans as indicated in Figure 1.

Using ImageJ [54], images acquired at the same X-ray tube potential but using different configurations (i.e., “original”, as in Figure 1(a), and “inverted”, as in Figure 1(b)) were averaged pixel by pixel. All the images had a matrix with a size of 1024 × 1024. The samples were kept as close to each other as possible during the scans to further compensate for the nonuniform intensity of the X-ray beam.

The same software was also utilized to measure the mean pixel intensity of each XCA in a square-shaped region-of-interest (ROI) with sides made of 50 pixels. These values were used to calculate the CNRs. The analysis was performed in triplicate.

#### 2.1.3. Digital Radiography

**Figure 2:**
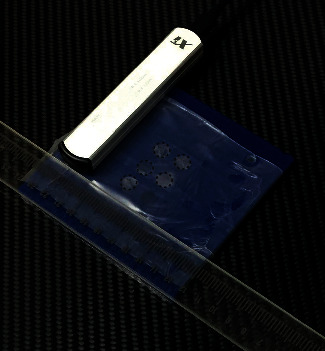
Sample holder. The sample holder, made of PMMA, was able to accommodate up to six XCA with a volume of 175 *µ*L. There was a distance of 0.5 cm between wells. The circumferences of the wells were delineated with dashed lines to make them more noticeable. The position of the ionization chamber was kept the same in all the exposures.

The XCA with a concentration of rhenium and iodine of 200 mM were imaged at 50, 81, and 121 kVp using a clinical X-ray tube camera (Multix X-ray, Siemens AG, Munich, Germany) with a digital radiography retrofit (DRX; New York, United States). A sample holder with six 200 *µ*L wells was fabricated with PMMA to image the solutions (Figure 2). For all the scans, a total of 175 *µ*L of each solution were pipetted into the wells. As in all previous experiments, water and air were used as controls.

The distance between the XCA and the X-ray tube focal spot was set to 102 cm. As per the manufacturer's specifications, the instrument's inherent filtration was 3.9 mm of aluminum. The exposures were carried out with varying thicknesses of additional filtration, from 0 to 1.5 mm of copper. In all the scans, a RAYSAFE Xi platinum (Fluke, United States) was placed on top of the samples as shown in Figure 2 to measure the incident air kerma. The settings of the scans are summarized in Table 3.

All the images had a matrix with a size of 2520 × 3032 and were processed on the digital system with the “Pattern” algorithm (a flat field algorithm). ImageJ was used to measure the mean pixel intensities of the samples in circular ROIs with a diameter of 5 pixels. These values were then used to calculate the CNRs. The analysis was performed in triplicate.

### 2.2. Investigation of the Absorbed Dose

Using as a reference the experimental setup described in Section 2.1.3, the depth absorbed dose profile and the average absorbed dose in a Solid Water® phantom (Best, Nashville, Tennessee, United States) were estimated utilizing Monte Carlo simulations implemented with Geant4 Application for Tomographic Emission (GATE) [55]. For simplicity, the phantom was simulated as a cuboid with length, width, and height of 30, 30, and 20 cm, respectively. The calculations were performed considering an inherent filtration of 1 mm of aluminum and 0.15 mm of beryllium. The distance between the phantom and the X-ray tube was set to 100 cm.

**Figure 3:**
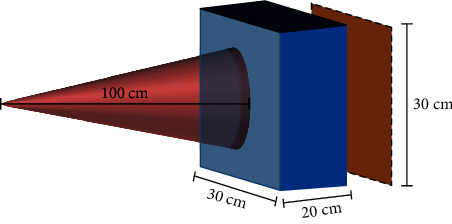
Geometry of Monte Carlo simulation. The schematic shows the X-ray beam (red), the Solid Water® phantom (blue), and the detector (brown). A FOV of 20 cm at the surface of the phantom was chosen. All the simulations were conducted assuming a total of 8.5 × 10^5^ photons in the transmitted X-ray beam.

The simulations were conducted at 50, 80, and 120 kVp in the absence of sources of additional filtration. Then, they were repeated at 120 kVp in the presence of 0.1, 0.3, 0.5, 1, and 1.5 mm thick copper filters. The X-ray spectra were modeled using experimental data acquired with the preclinical *µ*CT scanner (eXplore CT120, TriFoil Imaging; Chatsworth, USA) described in Section 2.1.1. For each exposure, the current was adjusted to simulate 8.5 × 10^5^ photons transmitted out of the phantom (i.e., reaching the detector). This value was defined based on the number of photons in the transmitted X-ray beam at 120 kVp with 0.5 mm of copper as additional filtration, also determined with Monte Carlo calculations. The detector was considered to have an efficiency of 100%. All the simulations were carried out assuming a static and divergent X-ray beam with a field of view (FOV) of 20 cm in diameter on the phantom's surface.  Figure 3 depicts a schematic of the geometry of the simulation.

### 2.3. Preparation of Rhenium-Doped Electrospun Scaffolds

A rhenium-doped scaffold made of polycaprolactone (PCL) was produced by electrospinning, a technique used to produce scaffolds constituted of nanofibers (NFs). A rhenium phosphinophenolate complex [ReOCl(MePO)_2_] was synthetized [56] and dissolved at a concentration of 2% w/v in a mixture of chloroform and methanol (70/30% v/v). Subsequently, 45 kDa PCL (Sigma Aldrich, Oakville, Ontario, Canada) was added at a concentration of 20% w/v and 2.5 mL of the solution was electrospun in a 10 mL syringe with a 20G 1/2” blunt needle using an electrospinner (Kato Tech Co., Ltd.; Kyoto, Japan). The electrospinning distance was set to 20 cm, the distance advancement rate to 0.07 mm·min^−1^, and the voltage to 25 kV. The system was maintained in a horizontal position. The collecting plate was wrapped in aluminum foil, which acted as conducting material, and it was rotated at a speed of 20 cm min^−1^. The rhenium-doped scaffold was collected on a 3 cm strip for ∼4 h. A rhenium-free scaffold (i.e., without the rhenium complex) was produced under the same experimental conditions.

To investigate their morphology at the nanoscale, 0.25 cm^2^ square-shaped samples of the scaffolds were imaged by scanning electron microscopy (SEM; SU3500, Hitachi; Chiyoda, Japan). The samples were mounted onto a specimen stub covered with an electrically conductive carbon-based adhesive disc. They were then sputter-coated with an 8 nm layer of iridium using a modular high vacuum coating system (EM MED020, Leica, Wetzlar, Germany) under reduced pressure (<5 Pa). The images were acquired at 15 kV with a magnification of 10,000x.

A rectangular-shaped sample of the rhenium-doped scaffold was incorporated onto the surface of a short section of a vascular catheter made of polyethylene (PE-50, ID = 0.05 cm and OD = 0.10 cm, where ID and OD stand for inner and outer diameters, respectively; Intramedic™, Becton Dickinson). The sample had a length of 1.5 cm and a width of approximately 2 mm, but it was actually constituted of eight layers, which facilitated handling and processing. The catheter was wrapped with the scaffold carefully but tightly. The scaffold/catheter system was then completely covered with aluminum foil and placed directly on the surface of a hot plate equipped with a temperature probe. The temperature was initially set to 55°C but it was slowly increased in 1°C increments until the scaffold melted, which occurred at 59°C. The catheter was removed from the hot plate and allowed to cool down. The same procedure was utilized to coat another section of the same catheter with the rhenium-free scaffold.

Following the same steps, the balloon in a percutaneous transluminal coronary angioplasty (PTCA) dilatation catheter (Long Cobra 40™, SciMed Life Systems, Inc.) was coated with the rhenium-doped and the rhenium-free scaffolds. The scaffolds were applied directly on top of the balloon, without air (i.e., not expanded).

The coated sections of the catheters were cut and placed inside microcentrifuge tubes for imaging by *μ*CT using a high-resolution specimen scanner (*μ*CT 100, SCANCO Medical, Brüttisellen, Switzerland). The samples were imaged at 90 kVp (200 *μ*A and 550 ms). According to the manufacturer's specifications, the only source of filtration in the scanner was the X-ray window, made of 0.15 mm of beryllium. The acquisition consisted of 1,000 projections. Using MicroView (Parallax Innovations, Ilderton, Canada), the data was reconstructed as a 3D image with a matrix size of 2048 × 2048 and a spatial resolution of 5 *μ*m.

## 3. Results and Discussion

### 3.1. Investigation of the Image Quality

#### 3.1.1. Micro-Computed Tomography

**Figure 4:**
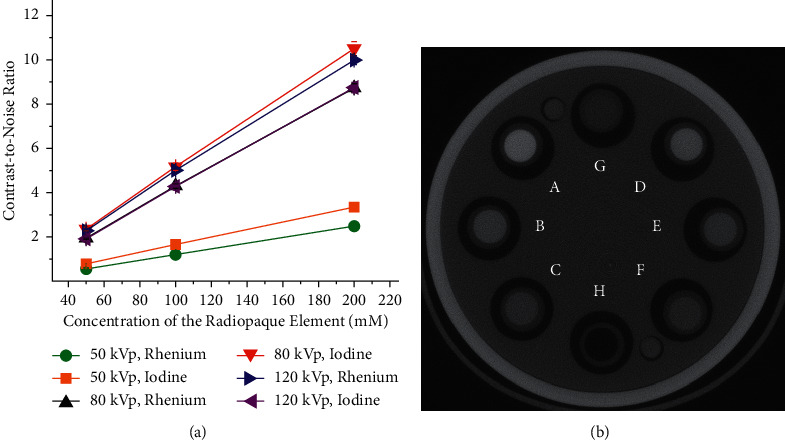
Evaluation of the contrast-to-noise ratio in micro-computed tomography. (a) The graph shows the relationship between the CNR and the concentration of the radiopaque element in the XCA. The scans were carried out at 50 kVp (10 mAs), 80 kVp (7.5 mAs), and 120 kVp (2.5 mAs) with a 0.6 mm thick copper filter, that is, exposures X01, X02, and X03, respectively (see Table 1). (b) Axial image acquired at 50 kVp showing the following XCA: I-XCA ((A) 200 mM, (B) 100 mM, and (C) 50 mM), Re-XCA ((D) 200 mM, (E) 100 mM, and (F) 50 mM), and controls ((G) water and (H) air). Attenuation: (A) 1,746 HU, (B) 863 HU, (C) 403 HU, (D) 1,297 HU, (E) 622 HU, (F) 285 HU, (G) −9.5, and (H) −994 HU. The scan corresponds to X01 (see Table 1). The XCA were imaged by *µ*CT.

A positive correlation was found between the CNR and the concentration of rhenium and iodine at each X-ray tube potential. That is, there is an improvement in contrast as the concentration of rhenium and iodine is increased from 50 to 200 mM. These findings, which are consistent with other studies [57–59], are depicted graphically in Figure 4(a) for the exposures performed with a 0.6 mm thick copper filter. A representative axial image of the samples acquired at 50 kVp is depicted in Figure 4(b). The attenuation, reported in HU, was calculated using the Hounsfield scale, which is a linear transformation of the linear X-ray attenuation coefficient that takes as a reference the attenuation of air and water [60].

**Figure 5:**
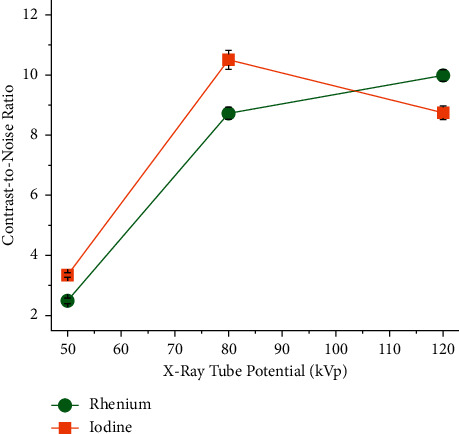
Effect of the X-ray tube potential on the contrast-to-noise ratio. The concentration of rhenium and iodine in the XCA was 200 mM. A 0.6 mm thick copper filter was used as additional filtration. The XCA were imaged by *µ*CT. Settings: 50 kVp (10 mAs), 80 kVp (5 mAs), and 120 kVp (2.5 mAs), that is, exposures X01, X02, and X03, respectively (see Table 1).

The quantitative VOI-based analysis showed that the CNRs of rhenium and iodine are dependent upon the X-ray tube potential. This is exemplified in Figure 5 for the scans conducted with a 0.6 mm thick copper filter. The I-XCA has a greater CNR than the Re-XCA at 50 and 80 kVp, but the Re-XCA has a higher CNR at 120 kVp.

**Figure 6:**
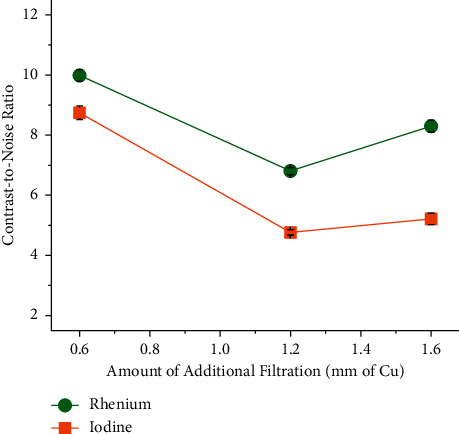
Effect of the amount of additional filtration on the contrast-to-noise ratio. The percent CNR improvement of rhenium over iodine is increased as the amount of additional filtration is increased. The concentration of rhenium and iodine in the XCA was 200 mM. The XCA were imaged by *µ*CT. All the scans were conducted at 120 kVp. They correspond to exposures X03, X04, and X05 (see Table 1).

**Figure 7:**
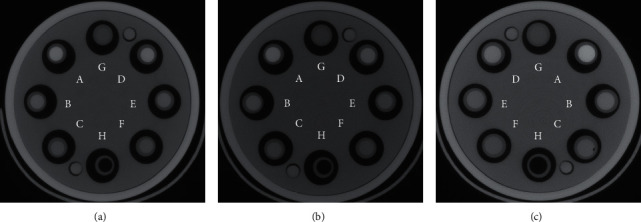
Micro-computed tomography imaging of rhenium and iodine. Representative axial images acquired at 120 kVp by *µ*CT with copper filters with the following thicknesses: (a) 0.6, (b) 1.2, and (c) 1.6 mm. The exposures correspond to X03, X04, and X05, respectively (see Table 1). The images depict the following XCA: Re-XCA ((A) 200 mM, (B) 100 mM, and (C) 50 mM), I-XCA ((D) 200 mM, (E) 100 mM, and (F) 50 mM), and controls ((G) water and (H) air). Attenuation: in (a), (A) 917 HU, (B) 460 HU, (C) 210 HU, (D) 803 HU, (E) 393 HU, (F) 176 HU, (G) 0, and (H) −998 HU. In (b), (A) 942 HU, (B) 473 HU, (C) 218 HU, (D) 661 HU, (E) 321 HU, (F) 138 HU, (G) 1, and (H) -996 HU. In (c), (A) 971 HU, (B) 486 HU, (C) 227 HU, (D) 610 HU, (E) 305 HU, (F) 137 HU, (G) 0, and (H) −998 HU.

Figure 6 shows the effect of the addition of further filters of copper on the CNRs of the XCA at 120 kVp. The difference in CNR is increased as the thickness of the copper filter is increased from 0.6 to 1.6 mm. For instance, the percent CNR improvement of rhenium over iodine is 14.2% when the thickness of the copper filter is 0.6 mm, but it is 59.1% when the thickness of the copper filter is 1.6 mm.  Figure 7 depicts axial images acquired in all of these scans.

**Figure 8:**
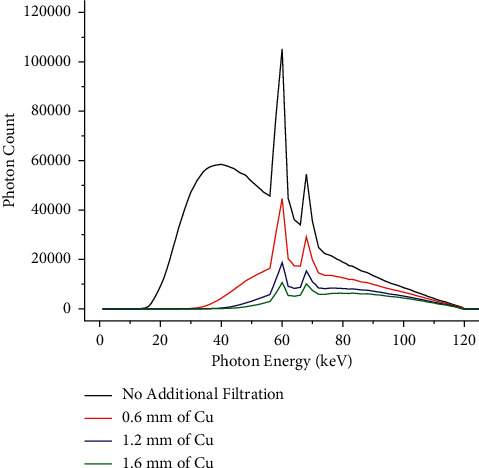
Effect of copper on the 120 kVp X-ray spectra. The image shows the 120 kVp X-ray spectra transmitted through copper filters with thicknesses up to 1.6 mm. The mean energy of each X-ray spectra is 39.8 keV when there is no additional filtration, 63.2 keV with a 0.6 mm thick copper filter, 67.8 keV with a 1.2 mm thick copper filter, and 69.5 keV with a 1.6 mm thick copper filter. The X-ray spectra were predicted using SpekCalc.

These results suggest that rhenium exhibits a kVp-dependent superiority in CNR (thus, in attenuation) over iodine. The greater attenuation of rhenium at 120 kVp is associated with the differential K-edge of rhenium and iodine. Based on data published by the National Institute of Standards and Technology (NIST), [61] the mass attenuation coefficient (i.e., the linear attenuation coefficient divided by the density, expressed in units of cm^2^ g^−1^) of iodine is higher than rhenium between 33.1 keV (iodine's K-edge) and 71.7 keV (rhenium's K-edge). However, the mass attenuation coefficient of rhenium is consistently higher than iodine above 71.7 keV; Figure 8 depicts the 120 kVp X-ray spectra simulated with SpekCalc, where it is observed that the X-ray beam produced in each scan was significantly hardened with the addition of copper filters. For instance, the mean energy of the 120 kVp X-ray beam was increased from 39.8 keV (without a copper filter) to 63.2, 67.8, and 69.5 keV (with 0.6, 1.2, and 1.6 mm thick copper filters, respectively). This suggests that the main factor contributing to the increased CNR of rhenium at 120 kVp is the removal of low keV photons, which are more likely to interact with iodine.

#### 3.1.2. Planar X-Ray Imaging

**Figure 9:**
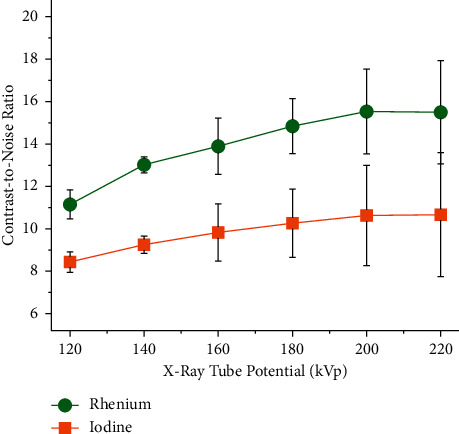
Effect of the X-ray tube potential on the contrast-to-noise ratio. The Re-XCA consistently displays a greater CNR than the I-XCA from 120 to 220 kVp. The concentration of rhenium and iodine in the XCA was 200 mM. The XCA were imaged by planar X-ray imaging using the SARRP. All the scans were carried out with a current of 50 *μ*A and 0.1 mm of copper as additional filtration, that is, exposures X06 to X11 (see Table 2).

The relationship between the CNR and the X-ray tube potential is depicted in Figure 9. The graph shows that the CNR of iodine at 220 kVp is 26.4% greater than at 120 kVp. This moderate improvement in CNR is associated with the overall increase in photon count at 220 kVp. Rhenium, on the other hand, shows an increase in CNR of 39.0%. The improvement in CNR of rhenium at 220 kVp is associated with the increase in the proportion of photons with energy above rhenium's K-edge. Furthermore, it was found that the difference in CNR between rhenium and iodine is increased when the X-ray tube potential is increased. For example, the percent CNR improvement of rhenium over iodine is 32.2% at 120 kVp, but it is 45.3% at 220 kVp.

#### 3.1.3. Digital Radiography

**Figure 10:**
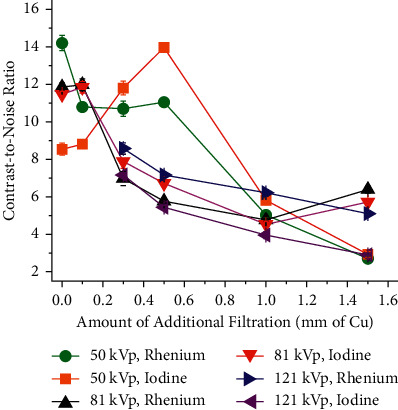
Evaluation of the contrast-to-noise ratio in digital radiography. The concentration of rhenium and iodine in the XCA was 200 mM. The XCA were imaged by digital radiography using a clinical X-ray camera (Multix X-ray, Siemens AG; Munich, Germany). The scans correspond to X12 to X23 and X26 to X29 (see Table 3). The images from exposures X24 and X25 were excluded from the analysis due to lack of contrast. As a consequence of the use of insufficient additional filtration in these two scans, the detector was saturated with photons when the current was 1.25 mA, which is the lowest current that can be selected in the instrument.

The variation in CNR at 50, 81, and 121 kVp as a function of the amount of additional filtration is graphically shown in Figure 10. The experiments conducted by *µ*CT showed that iodine exhibits a greater CNR than rhenium at 50 kVp (see Figure 5). Figure 10 shows that rhenium can exhibit a greater CNR than iodine at 50 kVp depending on the amount of additional filtration. At 50 kVp, rhenium has a higher CNR than iodine in two cases: (1) without any source of additional filtration (i.e., the only source of filtration is the instrument's inherent filtration, equivalent to 3.9 mm of aluminum) and (2) with a 0.1 mm thick copper filter. These differences in attenuation are caused by changes in the energy spectrum of the X-ray beam incident on the XCA.

Similar to the scans performed by *µ*CT and planar X-ray imaging, rhenium consistently has a greater CNR than iodine at 121 kVp. While the CNRs of both samples are decreased as the thickness of the copper filters is increased, the difference in CNR between rhenium and iodine is increased. For example, the percent CNR improvement of rhenium over iodine is 19.8% in the presence of a 0.3 mm thick copper filter, but it is 75.9%, almost four times higher, in the presence of a 1.5 mm thick copper filter.

### 3.2. Investigation of the Absorbed Dose

The incident air kerma and the mean energy of the X-ray beam, calculated from X-ray spectra predicted using SpekCalc, are summarized in Table 4.

**Figure 11:**
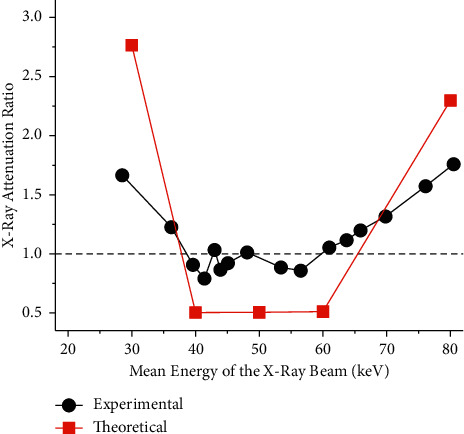
Rhenium-to-iodine X-ray attenuation ratio. The experimental curve was derived by dividing the mean pixel intensity of rhenium by the mean pixel intensity of iodine and dividing this value by the incident air kerma. The theoretical curve, on the other hand, was calculated from data reported by the NIST [61]. Basically, it represents the ratio of the mass X-ray attenuation coefficient of rhenium to the mass X-ray attenuation of iodine.

To compare the differential attenuation of rhenium and iodine across all the scans, we calculated a rhenium-to-iodine X-ray attenuation ratio. We divided the mean pixel intensity of rhenium by the mean pixel intensity of iodine and divided this quotient by the incident air kerma of each scan.  Figure 11 illustrates the relationship between the rhenium-to-iodine X-ray attenuation ratio, determined experimentally, and the mean energy of the X-ray beam. As a reference, a theoretical rhenium-to-iodine mass X-ray attenuation coefficient ratio was also calculated and plotted in Figure 11. Basically, when the rhenium-to-iodine X-ray attenuation ratio is above 1, rhenium outperforms iodine (i.e., rhenium is expected to have a higher attenuation and CNR), but when it is below 1, iodine outperforms rhenium (i.e., iodine is expected to have a higher attenuation and CNR). The deviation between the experimental and the theoretical curves might be due to noise in the images and scattering from the surrounding media.

A higher incident air kerma is expected when a larger X-ray tube potential is applied because photons with greater energy are produced. The incident air kerma, however, does not necessarily correlate with absorbed dose [62]. Using Monte Carlo, we assessed the differences in absorbed dose in 50, 80, and 120 kVp scans, similar to the ones described above.

**Figure 12:**
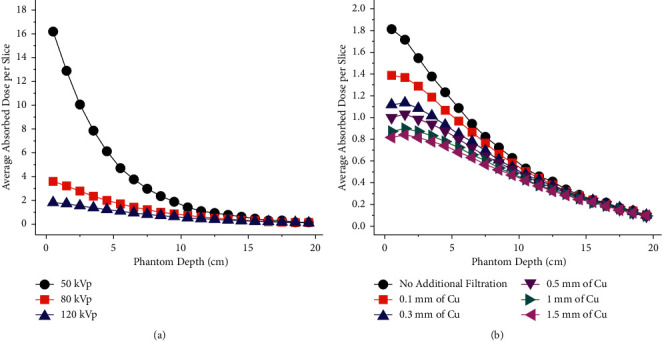
Depth absorbed dose profiles. Using Monte Carlo, the depth absorbed dose profiles were predicted for scans carried out (a) at 50, 80, and 120 kVp without additional filtration and (b) at 120 kVp with copper filters with thicknesses between 0.1 and 1.5 mm. The phantom was simulated as a 30 × 30 × 20 cm^3^ Solid Water® cuboid. The current was adjusted to simulate 8.5 × 10^5^ photons transmitted out of the phantom (i.e., reaching the detector).

Figure 12(a) depicts the change in the average absorbed dose per slice (with a thickness of 1 cm) as a function of the depth of the phantom for 50, 80, and 120 kVp scans with no additional filtration. At 50 kVp, significantly more radiation dose is deposited in the phantom. The relative average absorbed dose was found to be five times larger at 50 kVp than at 120 kVp. Also, while the difference is not as large, the relative average absorbed dose at 80 kVp is almost double that at 120 kVp. These values are summarized in Table 5.  Figure 12(b) shows that the relative absorbed dose is significantly reduced at 120 kVp when the amount of additional filtration is increased. For example, the relative average absorbed dose is reduced 37.6% after the addition of a 1.5 mm thick copper filter. The curves show that the highest deposition of radiation dose occurs within a couple of centimeters of the surface of the phantom.

### 3.3. Preparation of Rhenium-Doped Electrospun Scaffolds

**Figure 13:**
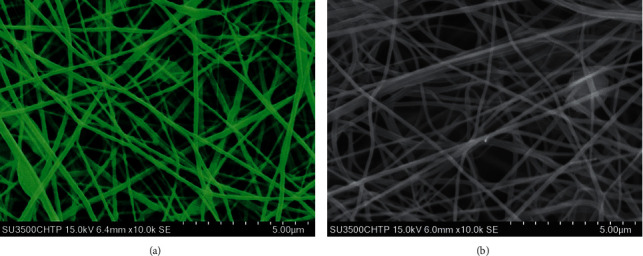
Morphology inspection of rhenium-doped and rhenium-free scaffolds. Images acquired by SEM are shown for (a) the rhenium-doped scaffold and (b) the rhenium-free scaffold. A pseudocolor was assigned to (a) to reflect the tint observed in the rhenium-doped scaffold.

SEM showed that both the rhenium-doped and the rhenium-free scaffolds were made of a smooth and continuous network of NFs (Figure 13).

**Figure 14:**
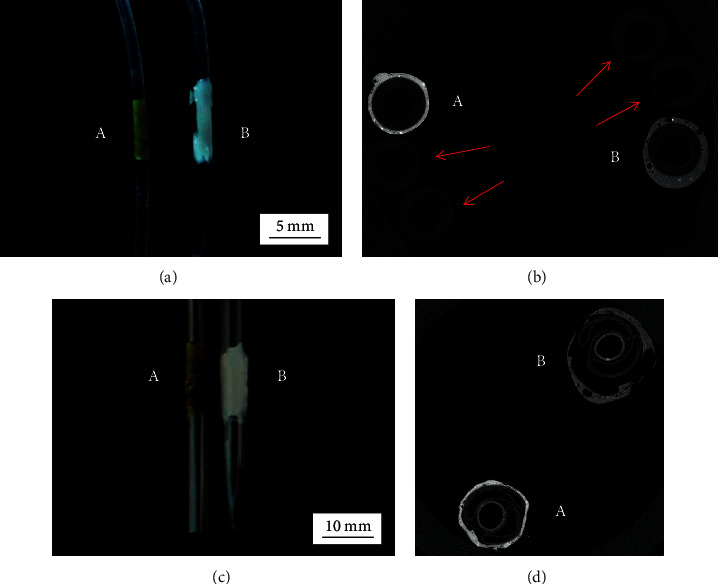
X-ray imaging of coated catheters. The images show (a, b) the vascular catheters and (c, d) the PTCA dilatation catheters coated with samples of (A) the rhenium-doped scaffold and (B) the rhenium-free scaffold. (b, d) Representative axial images acquired at 90 kVp (110 *μ*As). (b) The catheters in (a). (d) The catheters in (c). A significant improvement, in contrast, is observed with the rhenium-doped polymer-based coating. Four uncoated catheters are included in (b) (indicated with red arrows).

Figures 14(a) and 14(c) show images of the two catheters after coating them with samples of the rhenium-doped and the rhenium-free scaffolds. Figures 14(b) and 14(d) show representative *µ*CT axial images of the catheters, where it is seen that the rhenium-doped polymer-based coating has higher contrast than the rhenium-free polymer-based coating.

No variation in the contrast was observed across the rhenium-doped polymer-based coating. This was demonstrated quantitatively by calculating the distribution of grey values across the coatings using Image-Pro Plus 7 (Media Cybernetics, Rockville, USA), an image analysis software. A circle was drawn inside the coatings of both catheters in the *μ*CT axial images shown in Figures 14(b) and 14(d). The intensities of all the pixels in the circles were then calculated in grey values. This analysis was also performed in uncoated sections of the catheters.

**Figure 15:**
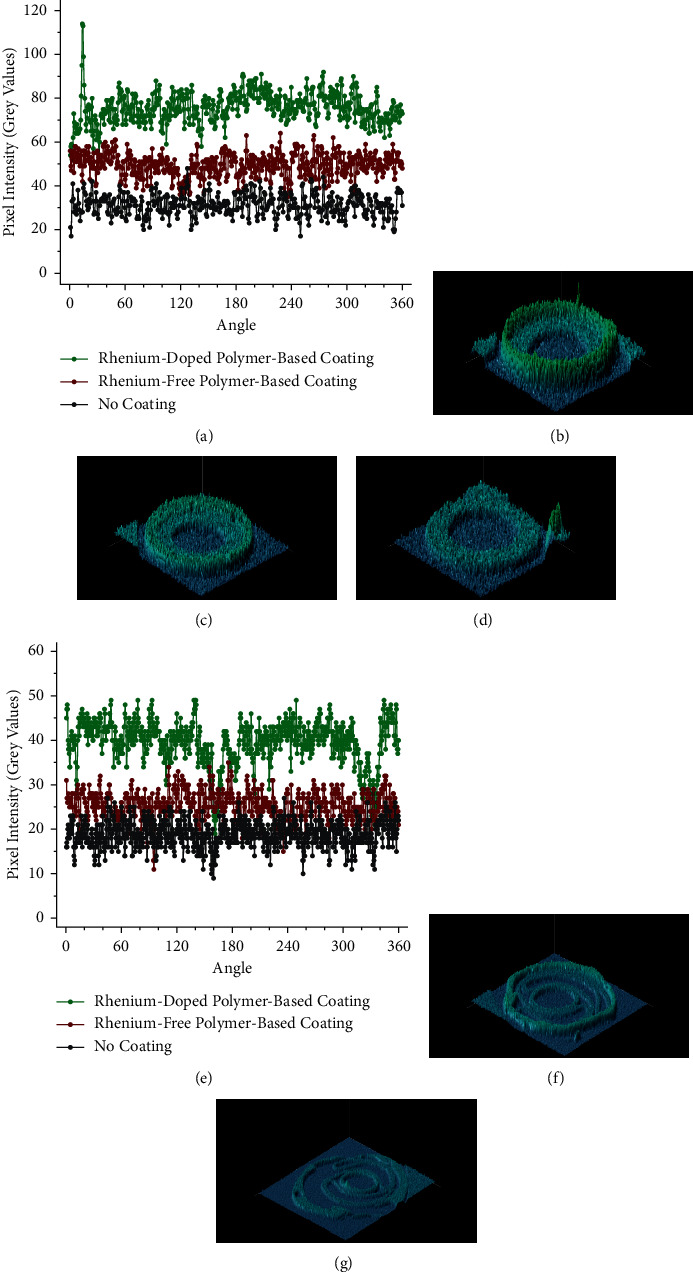
Pixel intensity analysis of the coatings. The pixel intensities were determined by measuring the grey values across circles drawn inside the coatings using Image-Pro Plus 7 (Media Cybernetics, Rockville, USA). The variation in pixel intensity across the coatings is shown in (a) for the vascular catheter and (e) for the PTCA dilatation catheter. 3D plots of the distribution of grey values are shown for the vascular catheter in (b), (c), and (d) and for the PTCA dilatation catheter in (f) and (g). Samples: (b, f) rhenium-doped polymer-based coating, (c, g) rhenium-free polymer-based coating, and (d) no coating.

The variation in the pixel intensities of the coatings is shown graphically in Figure 15(a) for the vascular catheter and Figure 15(e) for the PTCA dilatation catheter. Figure 15 also shows 3D plots of the distribution of grey values in the coatings, generated with the same software. The mean pixel intensities calculated for the coatings are reported in Table 6. For the vascular catheter, there is a 142% increase in pixel intensity when the catheter is coated with the rhenium-doped polymer-based coating (calculated relative to the pixel intensity of an uncoated section of the catheter). For the PTCA dilatation catheter, there is a 109% increase in pixel intensity (calculated relative to the pixel intensity of the balloon, without a coating). Furthermore, the standard deviations fall within 10% of the mean values, thus suggesting there is minimal variation in the pixel intensities of the coatings.

## 4. Conclusion

This study provided further evidence that rhenium exhibits a kVp-dependent superiority in CNR and attenuation over iodine. While there is typically a trade-off between absorbed dose and image quality, we showed that the absorbed dose is lower in scans where the CNR of rhenium is higher than that of iodine (specifically in scans over 120 kVp with the use of filtration). High kVp X-rays are commonly used for imaging average-sized and large patients, but noisy images are often acquired due to the suboptimal X-ray attenuation of iodine. The utilization of rhenium in high kVp X-rays has the potential of enhancing contrast while minimizing absorbed dose.

We also showed that rhenium-doped electrospun scaffolds can be used to coat catheters, thus making them truly radiopaque. Making catheters radiopaque may allow physicians to place them inside the body more rapidly and precisely, potentially even with minimal or without the administration of I-XCA. This may also have applications to enhance contrast in guidewires and micro-guidewires used in pediatric applications.

Future work revolves around the development of novel, biodegradable Re-XCA and investigation of their *in vitro* and *in vivo* toxicity, as well as their biodistribution. For instance, rhenium-doped microspheres and NP have the potential of replacing clinically used I-XCA, thus increasing the contrast and the blood circulation time.

## Figures and Tables

**Table 1:** Settings for micro-computed tomography.

Scan ID	X-ray tube potential (kVp)	Amount of additional filtration (mm of Cu)	Current (mA)	Exposure time (ms)	Current·exposure time product (mAs)
X01	50	0.6	80	125	10
X02	80	0.6	60	125	7.5
X03	120	0.6	40	63	2.5
X04	120	1.2	40	63	2.5
X05	120	1.6	40	125	5

The XCA were imaged at 50, 80, and 120 kVp using a preclinical *µ*CT scanner. The current and the exposure time were selected to avoid saturation of the detector. The spatial resolution of the images after reconstruction was 50 *μ*m.

**Table 2:** Settings for planar X-ray imaging.

Scan ID	X-ray tube potential (kVp)	Amount of additional filtration (mm of Cu)	Current (mA)
X06	120	0.1	0.5
X07	140	0.1	0.5
X08	160	0.1	0.5
X09	180	0.1	0.5
X10	200	0.1	0.5
X11	220	0.1	0.5

The XCA were imaged between 120 and 220 kVp using the SAARP.

**Table 3:** Settings for digital radiography.

Exposure ID	X-ray tube potential (kVp)	Amount of additional filtration (mm of Cu)	Current (mA)
X12	50	0	1.25
X13	50	0.1	2.5
X14	50	0.3	10
X15	50	0.5	36
X16	50	1	71
X17	50	1.5	71
X18	81	0	1.25
X19	81	0.1	1.6
X20	81	0.3	1.6
X21	81	0.5	2.2
X22	81	1	4
X23	81	1.5	4
X24	121	0	1.25
X25	121	0.1	1.25
X26	121	0.3	1.25
X27	121	0.5	1.25
X28	121	1	1.25
X29	121	1.5	1.25

The Re- and I-XCA were imaged at 50, 81, and 121 kVp using a clinical X-ray camera. The current was chosen to avoid saturation of the detector.

**Table 4:** Measurement of the incident air kerma and the mean energy of the X-ray beam.

Scan ID	X-ray tube potential (kVp)	Amount of additional filtration (mm of Cu)	Incident air kerma (*μ*Gy)	Mean energy (keV)
X17	50	0	14.9 + 0.1	28.5
X18	50	0.1	10.5	36.2
X19	50	0.3	11.2	39.6
X20	50	0.5	15.4	41.4
X21	50	1	4.2	43.9
X22	50	1.5	1.3	45.1
X23	81	0	49.3 + 0.2	43.1
X24	81	0.1	31.9 + 0.6	48.1
X25	81	0.3	15.4 + 0.1	53.4
X26	81	0.5	12.5	56.5
X27	81	1	9.3	61.0
X28	81	1.5	21.2 + 0.1	63.7
X29	121	0	114.9 + 0.2	53.9
X30	121	0.1	74.0 + 0.2	59.7
X31	121	0.3	46.0 + 0.1	65.9
X32	121	0.5	33.4 + 0.1	69.8
X33	121	1	19.7 + 0.1	76.1
X34	121	1.5	11.7 + 0.1	80.5

The incident air kerma is reported as the mean of three independent measurements. The standard deviation is zero in cases where it is not reported.

**Table 5:** Relative average absorbed dose.

X-ray tube potential (kVp)	Amount of additional filtration (mm of Cu)	Mean energy (keV)	Relative current	Relative average absorbed dose
50	0	31.8	10.2	7.0
80	0	42.4	2.8	2.2
120	0	53.7	1.6	1.4
120	0.1	59.0	1.3	1.2
120	0.3	64.9	1.1	1.1
120	0.5	68.1	1.0	1.0
120	1	74.1	0.9	0.9
120	1.5	78.4	0.8	0.9

Using Monte Carlo, the average absorbed dose in a 30 × 30 × 20 cm^3^ Solid Water® cuboid was calculated at 50, 80, and 120 kVp. The effect of the addition of copper filters with thicknesses between 0.1 and 1.5 mm was also investigated. The average absorbed dose was considered to be 1 at 120 kVp in the presence of a 0.5 mm thick copper filter. The table also shows the mean energy of the X-ray beam incident on the phantom, which is similar to the experimental values within 10%. The relative current is the scaling factor utilized to equalize the number of photons in the transmitted X-ray beam, which was set to 8.5 × 10^5^ (i.e., the number of photons produced at 120 kVp in the presence of a 0.5 mm thick copper filter).

**Table 6:** Mean pixel intensity of the coatings.

Structure	Mean pixel intensity	
	Vascular catheter	PTCA dilatation catheter
Rhenium-doped polymer-based coating	75.5 + 7.1	39.5 + 5.0
Rhenium-free polymer-based coating	49.5 + 5.2	25.4 + 3.2
No coating	31.3 + 5.0	18.9 + 2.3

The mean pixel intensity of each structure was calculated by taking the average of all the grey values (see Figure 15). For the uncoated sections, the analysis was performed on the plastic for the vascular catheter and the balloon for the PTCA dilatation catheter.

## Data Availability

The data used to support the findings of this study are available from the corresponding author upon request.
